# The Mediating and Moderating Role of Social–Emotional Skills in the Relationship between Sports Participation and Test Anxiety

**DOI:** 10.3390/bs14060512

**Published:** 2024-06-20

**Authors:** Kun Wang, Jiali Qian

**Affiliations:** Department of Physical Education, Shanghai Jiao Tong University, 210 Guangming Hall, NO. 800 Dongchuan Street, Minhang District, Shanghai 200240, China; wangkunz@sjtu.edu.cn

**Keywords:** social–emotional skills, sports participation, test anxiety, mediation, moderation

## Abstract

Purpose: Test anxiety is a prevalent issue among adolescents, prompting a need for effective coping mechanisms. Participation in sports, which is gaining recognition for its crucial role in alleviating test anxiety, may be effective due to its association with social–emotional skills. Moreover, students with diverse levels of social–emotional skills not only experience enjoyment in sports differently but also perceive test anxiety uniquely, leading to varying interpretations of the relationships between them. Due to the lack of direct evidence, therefore, this study aimed to explore the intricate relationships among sports participation, test anxiety, and social–emotional skills. Methods: Utilizing OECD data from 61,010 participants across 10 locations, all variable measurements were collected through the Survey on Social and Emotional Skills (SSES). Results: The results indicated that social–emotional skills mediated and moderated the relationship between sports participation and test anxiety. However, only lower- and medium-level social–emotional skills significantly weakened the negative correlation between sports participation and test anxiety. Conclusion: Social–emotional skills serve a dual function in the relationship between sports participation and test anxiety. Not only do they elucidate why sports participation can alleviate test anxiety, but they also act as regulators, moderating the extent of this alleviation. These findings provide valuable insights for educational interventions, underscoring the importance of sports participation and the cultivation of social–emotional skills in mitigating test anxiety.

## 1. Introduction

In contemporary education, the pervasiveness of tests and their significant implications at all educational levels profoundly impact adolescents, leading to more and more adolescents with test-related anxiety [1,2]. While a certain degree of test anxiety can induce heightened alertness, excessive anxiety associated with tests may give rise to a spectrum of adverse emotional, physical, and cognitive symptoms [3], detrimentally impacting their overall well-being [4,5].

The anxiety associated with testing may stem not only from cognitive abilities and mastery of knowledge but also from the underdevelopment of essential non-cognitive psychological factors [6]. This anxiety is often observed in students who fear failing their tests [7], arising from various factors such as revision workloads, teachers’ attitudes towards tests and outcomes, and the results of mock examinations [7]. Test anxiety can manifest before, during, or after a test [8]. This study focuses primarily on pre-test anxiety, as it occurs at the onset and can significantly impact subsequent performance. By alleviating anxiety at an early stage, students can improve their test preparation and experience reduced anxiety during the test, ultimately leading to better overall results.

Addressing these challenges, some educators have developed psychological interventions to reduce test anxiety [9]. Additionally, as advocated for by the World Health Organization (WHO) [10], sports participation, as a non-invasive and easily accessible method, effectively alleviates anxiety by aiding individuals in managing and coping with their anxious emotions [11,12], including test anxiety [13,14], enhancing their well-being [15,16]. A meta-analysis revealed that engaging in at least twenty minutes of aerobic exercise and sports two to three times a week for a minimum of four weeks was necessary for significant test anxiety reduction [13].

The underlying mechanisms of this recommendation are multiple. Firstly, it may be linked to biological factors, such as the enhanced synthesis and release of neurotransmitters and neurotrophic factors [17,18,19], which aid in emotional regulation. Additionally, there are some other key mechanisms for anxiety reduction. One is the perception of anxiety. The interpretation of anxiety significantly influences how individuals assess challenging events and situations [20]. Participation in sports can help individuals reinterpret their perception of anxiety, enabling them to view anxiety from a more positive perspective, as surmountable difficulties rather than insurmountable burdens. As some top athletes stated, while anxiety responses can be demanding, symptoms perceived to be under control had facilitative consequences for performance [21], with various coping strategies tailored to their specific contexts [22]. Moreover, regular sports strengthen resilience and coping abilities, enhancing confidence and the capacity to handle anxiety. Individuals engaged in competitive sports often encounter significant anxiety, characterized by intense arousal as well as various physiological and emotional changes during competitions [23,24]. The heightened anxiety experienced in sports scenarios can make anxiety in other situations seem less intimidating by comparison. Furthermore, participation in sports communities provides crucial opportunities for social support, which plays a significant role in helping individuals cope with anxiety. Some adolescents, when confronted with anxiety, may take more proactive steps to manage it, including sharing problems and seeking support [25,26]. Engaging in team sports specifically offers social opportunities that are not only enjoyable but also serve as anxiety-relieving activities [27]. These potential influencing factors involve various non-cognitive abilities, also referred to as social–emotional skills.

Social–emotional skills refer to the capabilities that can regulate one’s thoughts, emotions, and behaviors [28]. Building on existing research, the Organization for Economic Co-operation and Development (OECD) has developed a comprehensive framework for these skills, based on the “Big Five” personality traits [28]. Five domains, including task performance, emotional regulation, collaboration, open-mindedness, and engaging with others, as well as two additional indices, lead to a multifaceted structure with a total of 17 skills [29,30].

Social–emotional skills are essential for individuals to effectively navigate life challenges [30], including anxiety over tests. A semester-long Social–Emotional Learning (SEL) program was implemented in statistics classes at two different institutions. Forty-six students participated in weekly activities designed to develop SEL-based skills and mindsets, with the aim of reducing anxiety. Significant improvements were noted in their ability to view stressors as positive challenges rather than threats. Furthermore, they showed an enhancement in identifying resources available for managing stress and anxiety, along with a decrease in math anxiety [31]. Additionally, a mixed-method study evaluated the effectiveness of an SEL intervention on student behavioral changes, including test anxiety. Qualitatively, it revealed that students developed new SEL strategies to more effectively handle stressful test situations [32].

Social–emotional skills are malleable [30], and physical education or participation in sports are important means of developing social–emotional skills [33]. Various game-based programs have been proposed for this purpose, including the Fun FRIENDS Program. In this program, 263 preschool children aged four to six were randomly assigned either to the Fun FRIENDS Program group or a control group. After 12 months, the intervention group showed significant improvements in social–emotional skills compared to the control group [34]. Similar outcomes were observed in a yoga intervention aimed at enhancing socio-emotional skills [35]. Additionally, a 30-week case study was conducted to investigate the experiences of children with Social and Emotional Learning (SEL) in Sports-based Youth Development (SBYD) programs. This study involved seventeen middle school boys (N  =  17) from low-income families who were enrolled in SBYD programs, highlighting how SBYD fosters their social–emotional skills [36].

Considering the enhancement of social–emotional skills through physical education and sports participation, and the role of social–emotional skills in alleviating test anxiety, it appears that social–emotional skills may serve as an important mediating mechanism in interpreting the relationship between sports participation and test anxiety. That is, social–emotional skills could be a key mediator between sports participation and test anxiety. Previous studies have directly or indirectly mentioned certain social–emotional skills, or their explanatory roles in sports-related [37] or test anxiety-related [38] contexts. However, research that utilizes a systematic and comprehensive framework like that of the OECD to understand these issues remains relatively limited. Additionally, the OECD provides extensive global data, offering robust support for this research to explore this relationship firmly.

Nevertheless, it is interesting to note that anxiety coping strategies vary based on situational and personal factors [25,26,39]. This suggests that individuals with diverse personality temperaments or varying levels of social–emotional skills, built upon personality, can influence the perception of anxiety, including test anxiety, as well as perspectives on the relationship between sports participation and test anxiety. There might be a moderating role of socio-emotional skills in the correlation between sports participation and test anxiety. Unfortunately, direct evidence supporting this is currently limited as well.

In summary, social–emotional skills are assumed to serve as an important explanatory mediator for how sports participation can alleviate test anxiety. Due to the malleable nature of social–emotional skills, their role in the relationship between sports participation and test anxiety may vary, resulting in different effects on this relationship. This highlights the importance and necessity of considering the dynamic nature of social–emotional skills when examining their roles in the relationship between sports participation and test anxiety.

Previous research has not explicitly delineated the dynamic connection between sports participation, test anxiety, and social–emotional skills. Therefore, to address gaps in existing research, this study aims to explore the complex relationship among them, laying the groundwork for theoretical advancement and practical applications. Specifically, objective 1 investigates whether social–emotional skills mediate the relationship between sports participation and test anxiety. This objective seeks to determine if social–emotional skills can explain how sports participation helps alleviate test anxiety. Objective 2 explores whether social–emotional skills moderate the relationship between sports participation and test anxiety. This involves examining if the level of social–emotional skills influences the strength or direction of the relationship between sports participation and test anxiety.

**Figure 1:**
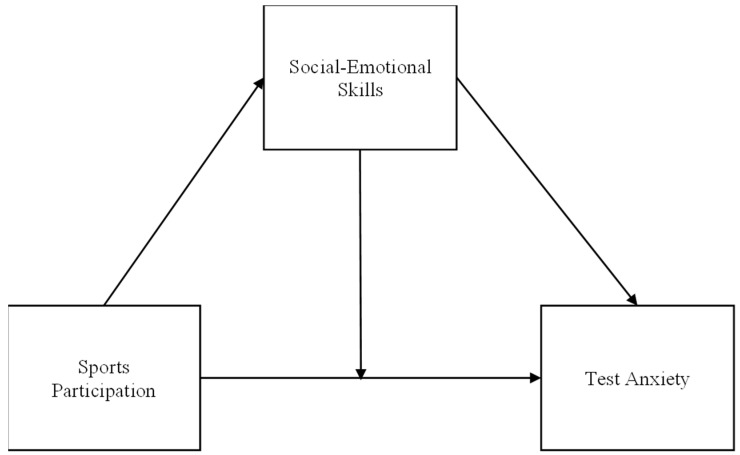
Moderated mediation model of test anxiety, social–emotional skills, and sports participation. Note: For simplicity, control variables are omitted.

This study proposed the open hypothesis based on the existing research, and constructed a mediation model and a moderation model to demonstrate this complexity (Figure 1). In the models, sports participation serves as the independent variable, and test anxiety is the dependent variable. Social–emotional skills not only play a mediating role but also a moderating role in the relationship between sports participation and test anxiety. The hypothesis posits the following:

**H1.** *Social–emotional skills mediate the relationship between sports participation and test anxiety.*

**H2.** *Social–emotional skills moderate the relationship between sports participation and test anxiety.*

## 2. Methods

### 2.1. Participants

The data, obtained from the OECD database [29], provide a comprehensive overview of diverse populations and socio-economic conditions from various global locations. The dataset includes 61,010 participants, though it is noteworthy that there are missing values in some demographic variables. It consists of two age groups, the older cohort (15-year-olds, N = 29,798) and the younger cohort (10-year-olds, N = 31,187). The gender distribution is composed of 29,863 males and 30,635 females. Regarding socio-economic status, the mean (standard deviation) is 0.226 (0.986) within a range of −3.75 to 3.75, covering 59,583 participants in total. The dataset includes contributions from ten global locations: Bogota, Colombia (N = 6771); Daegu, Korea (N = 6334); Helsinki, Finland (N = 5482); Houston, Texas, United States (N = 6434); Istanbul, Turkey (N = 5869); Manizales, Colombia (N = 6757); Moscow, Russian Federation (N = 6792); Ottawa, Canada (N = 5440); Sintra, Portugal (N = 3860); and Suzhou, People’s Republic of China (N = 7246). The details are in Table 1.

### 2.2. Measure

Measurements and calculations for all variables are sourced from the OECD. Detailed information is extensively elucidated on the OECD official website, and in technical reports [29], encompassing specific questions, weighting methods, calculation procedures, and the final computed scores.

Social–emotional skills were gauged using the Survey of Social and Emotional Skills (SSES). Each of the 15 skills was evaluated through 8 distinct questions, culminating in a total of 120 items. Two additional indices—self-efficacy and achievement motivation—were calculated based on other skills. Responses were recorded on a 5-point Likert scale, ranging from strong disagreement to strong agreement. Positive statements were scored from 0 to 4, while reverse scoring was used for negative statements. Weights were calculated based on demographics, and the final scores for each skill were calculated by the OECD. The higher the scores, the more developed the social–emotional skills. A comprehensive score for social–emotional skills was employed, ensuring a holistic summary of the skills. To address scale disparities, particularly in the additional indices, scores were standardized into Z-scores before aggregation. The reliability of the scales was confirmed, with Cronbach’s alpha surpassing 0.71.

Test anxiety was measured using the items STQM04201-STQM04203 from the SSES [29]. It should be noted that these items were part of the standardized OECD SSES dataset. Students rated their anxiety levels about tests on a spectrum from strongly disagree to strongly agree, scored from 1 to 5. The questions are as follows:

- I often worry that it will be difficult for me taking a test;
- Even if I’m well prepared for a test, I feel very anxious;
- I get very tense when I study for a test.

The final scores indicated the intensity of test anxiety, with higher scores indicating more intense test anxiety. The Cronbach’s alpha of 0.81 across sites indicates reliability. To ensure consistency in measurement scales, standardization was applied to the test anxiety.

Participation in sports outside school was determined through item STQM04301 in the SSES, titled “Participate outside school: sports” [29]. This item is a part of the standardized OECD SSES dataset. Responses were coded with 1 for non-participation and 2 for participation, with other responses considered missing.

### 2.3. Statistics Analysis

The initial dataset included 61,010 data. A thorough descriptive analysis of social–emotional skills and test anxiety revealed notable skewness and the presence of outliers. Then, data outside the 1st and 99th percentile thresholds in each group were classified as outliers and subsequently excluded. Missing values from raw data and identified outliers were treated through multiple imputations performed 20 times with 50 iterations each, utilizing the Predictive Mean Matching (PMM) method. This refinement resulted in 60,985 valid entries and 25 blank entries in the dataset. Dummy coding was implemented for sports participation and demographics, with missing data incorporated into the analysis as absent values.

Following basic descriptive statistics and Pearson correlation analysis, a mediation analysis was conducted using PROCESS model 4 [40] to explore the mediating role of social–emotional skills in the relationship between sports participation and test anxiety. Demographics were treated as covariates, based on the influence of them on the variables [41]. The mediation effect was assessed using bootstrapping (5000 iterations), with significance indicated by a 95% confidence interval that did not include zero. Next, moderated mediation was examined using PROCESS model 1 [40] to determine if social–emotional skills moderated the relationship between sports participation and test anxiety. The covariates were controlled, and social–emotional skills, the moderating variable, were centered. The interaction between social–emotional skills and sports participation, was examined. Significant interactions indicated a valid moderating effect. Finally, a simple slope analysis was conducted for the interaction effects. Sports participation was categorized into yes and no groups, while social–emotional skills were divided into low, medium, and high groups based on the mean minus one standard deviation (M − SD), the mean (M), and the mean plus one standard deviation (M + SD), respectively. Statistical analyses were conducted using SPSS 26.0 and Process 4.3, setting the significance level at 95%.

## 3. Results

### 3.1. Descriptive Statistics and Correlations

The means, standard deviations, and correlation coefficients of test anxiety, social–emotional skills, and sports participation are presented in Table 2. All correlation coefficients were statistically significant (*p* < 0.001). Sports participation showed a positive correlation with social–emotional skills, and a negative correlation with test anxiety. Social–emotional skills were negatively correlated with test anxiety.

### 3.2. Test of Mediation

**Figure 2:**
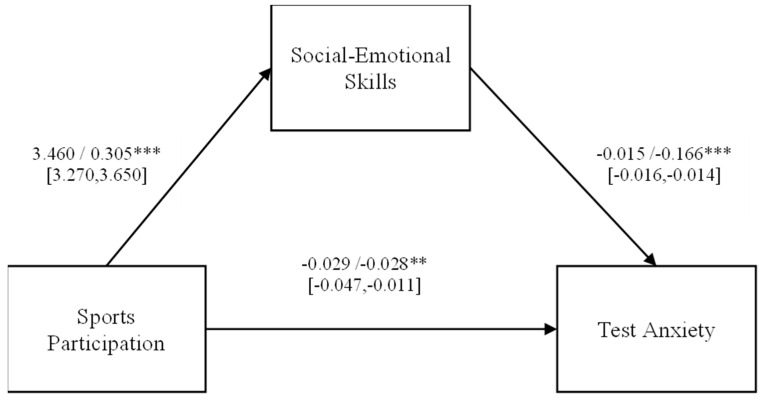
Mediation model of social–emotional skills. Note: The figures are unstandardized/standardized [LLCI, ULCI]. Covariates: gender, cohort, socio-economic status, site. ** *p* < 0.01; *** *p* < 0.001.

Controlling for demographic variables, the total effect of engaging in sports on test anxiety was examined, revealing a significant path coefficient (*β* = −0.081, *t* = −8.803, *p* < 0.001, 95%CI = [−0.099, −0.063]). Subsequently, after adding social–emotional skills as the mediating variable, new path coefficients and the bootstrap 95% confidence interval were obtained, as shown in Figure 2. All path coefficients were statistically significant (*p* < 0.001). The confidence interval of the indirect effect, [−0.056, −0.048], does not include zero, indicating the existence of a mediation effect by social–emotional skills, which accounts for 64.20% of the total effect (as detailed in Table 3).

### 3.3. Test of Moderation

The moderating effect of social–emotional skills was further explored in the relationship between sports participation and test anxiety (see Table 4). The interaction between social–emotional skills and sports participation (X × W) on test anxiety was significant (*β* = −0.002, *t* = 2.649, *p* = 0.008, 95%CI = [0.0005, 0.004]), demonstrating that social–emotional skills moderated the relationship between sports participation and test anxiety.

**Figure 3:**
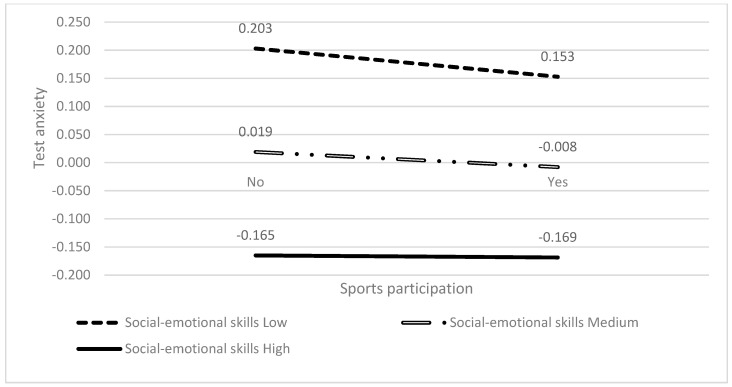
The moderating role of social–emotional skills on sports participation and test anxiety.

A simple slope analysis was conducted. The predictive effects of sports participation on test anxiety (Figure 3 and Table 5) were analyzed for high (M + SD), medium (M), and low (M-SD) levels of social–emotional skills, and showed the existence of moderation. Specifically, the analysis revealed a change in the negative correlation between sports participation and test anxiety at varying levels of social–emotional skills. Among students with lower levels of social–emotional skills, a significant association was found between participation in sports and reduced levels of test anxiety (bsimple = −0.050, *t* = −4.134, *p* < 0.001). For those with medium levels of social–emotional skills, the same trend persisted, as sports participation also significantly correlated with decreased test anxiety (bsimple = −0.027, *t* = −2.934, *p* = 0.003). For students with high social–emotional skills, a relationship between sports participation and reduced test anxiety levels was observed but did not reach statistical significance (bsimple = −0.004, *t* = −0.277, *p* = 0.782). There was a significant attenuation of the negative correlation between sports participation and test anxiety at lower and medium levels of social–emotional skills, with no significant effect observed at higher levels of social–emotional skills.

## 4. Discussion

The purpose of this study is to explore the relationship between sports participation, test anxiety, and social–emotional skills. There is a complex relationship among them; however, few studies have explored it in depth. In addition to confirming the established negative relationship between sports participation and test anxiety, this study also revealed the mediating and moderating roles of social–emotional skills in the aforementioned relationship.

The mediation analysis revealed that social–emotional skills serve as a mediator between sports participation and test anxiety. This suggests that engaging in sports alleviates test anxiety by enhancing social–emotional skills. Consistent with prior research, sports participation is associated with enhanced social–emotional skills [42,43,44]. Sports teach soft skills such as following orders, leadership, teamwork, performing in regulated systems, and socializing. These soft skills are challenging to acquire in the classroom but can be effectively developed in sports settings, enhancing positive or mitigating negative educational outcomes [45].

However, previous studies have only related to partial social–emotional skills [46,47] or cognitive aspects [48], and rarely used such a systematic and comprehensive framework to validate this issue with a large dataset. The OECD’s social–emotional skill survey database provides the necessary conditions to explore this issue thoroughly. According to the OECD classification, social–emotional skills can offer specific assistance in alleviating test anxiety. Task performance enables individuals to plan their exam preparation systematically, demonstrating greater resilience when facing academic challenges. Open-mindedness encourages individuals to embrace new learning methods and strategies, enhancing learning flexibility. It enables individuals to adapt to changes in academic disciplines and cope with diverse academic challenges, reducing resultant anxiety. Emotional regulation empowers students to control test-related nervousness and anxiety skillfully. By recognizing their emotional states and employing calming techniques, students can alleviate the psychological anxiety associated with tests. Engaging with others and collaboration enhance the ability to communicate and seek support. Students sharing study resources with peers or seeking assistance from teachers enhances learning efficiency and reduces anxiety arising from academic challenges. Furthermore, an increased sense of self-efficacy and the establishment of reasonable motivational goals can strengthen students’ confidence in their abilities, thereby reducing fears and anxieties related to tests.

It is important to note that social–emotional skills serve as a partial mediator in the relationship. This indicates that while social–emotional skills contribute to explaining how sports participation alleviates test anxiety, they are not the sole explanatory factor. Besides social–emotional skills, there may be other contributing factors. The existence of a direct effect aligns with research affirming that sports participation can directly help reduce test anxiety physiologically [17,18,19]. Additionally, the partial mediating effect aligns with the notion that the mechanisms through which sports produce anxiety may involve a combination of physiological and psychological factors [49].

Additionally, social–emotional skills can moderate the relationship between sports participation and test anxiety. Individuals’ actions are profoundly shaped by self-perception and the meanings attributed to ideas, inflecting how backgrounds mold their understanding of sport and its function [50]. Therefore, the level of social–emotional skills can influence the understanding of sports as well as the relationship between sports and test anxiety. Previous studies often used demographic variables as moderating factors to explore variations. However, in this study, we directly used different levels of social–emotional skills as moderating factors. This approach allowed us to adopt a more comprehensive perspective and a more targeted approach to our research question, leading to a nuanced understanding of the role of social–emotional skills in the relationship between sports participation and test anxiety.

The results showed that, at lower levels of social–emotional skills, engagement in sports has a more pronounced effect in reducing test anxiety within this group. For individuals with moderate social–emotional skills, physical exercise and sports remain effective in alleviating test anxiety, albeit with a reduced impact compared to those with lower skills. However, at higher levels of social–emotional skills, the mitigating effect of physical exercise on the relationship with test anxiety becomes statistically insignificant. This implies that it is necessary to consider the varying perspectives of students with different levels of social–emotional skills regarding sports and their functions. For students with low social–emotional skills, who might lack effective emotion regulation and anxiety management skills, engaging in sports serves as a vital method to process test anxiety. Engaging in sports and exercise induces the release of endorphins and other neurotransmitters that uplift mood and diminish anxiety. It also provides an opportunity to learn and practice test anxiety management skills, including teamwork, goal setting, and coping with failure. Consequently, sports may prove more effective in reducing test anxiety for students in this group. In contrast, students with higher social–emotional skills typically possess well-developed emotion regulation and coping strategies for test anxiety. For them, sports might serve more as a means to other functions rather than a primary coping mechanism for test anxiety. While sports remain beneficial for this group, their impact in alleviating test anxiety may not be as substantial as observed among students with lower levels of social–emotional skills. All in all, social–emotional skills influence the alleviating effect of sports participation on test anxiety among students at different levels. This should be taken into account when designing sports-related educational interventions to provide more personalized support and resources for different groups of students.

In summary, a complex interplay exists among sports participation, test anxiety, and social–emotional skills. Sports participation not only directly alleviates test anxiety but also enhances social–emotional skills, consequently mitigating test anxiety. The efficacy of sports participation in alleviating test anxiety is more pronounced in students with lower social–emotional skills. This may be attributed to the fact that, as social–emotional skills improve, students acquire the capacity to employ available coping strategies to alleviate test anxiety.

The findings enrich theoretical models concerning the complex relationships among sports participation, social–emotional skills, and test anxiety. By revealing social–emotional skills as both a partial mediator and moderator in this relationship, this study expands the understanding of how non-cognitive abilities interact with cognitive processes to influence emotional experiences. This insight can inform more nuanced theories of emotional regulation and psychological well-being, emphasizing the importance of integrating cognitive and non-cognitive aspects in psychological research. Additionally, the role of sports participation is highlighted as a crucial factor in developing social–emotional skills and managing test anxiety, further supporting its inclusion in theoretical frameworks related to adolescent mental health.

The practical implications of this study underscore the role of sports and social–emotional skills in promoting mental health among adolescents, particularly in managing and alleviating test anxiety. Social–emotional skills can mediate the relationship between sports participation and test anxiety and can also moderate this relationship. The results provide a new perspective for designing effective educational and psychological interventions, highlighting the importance of sports participation and the development of social–emotional skills in managing test anxiety for adolescents. Firstly, this study yields important insights for schools and education policymakers, emphasizing the significance of sports within the educational context. It demonstrates that sports not only contribute to students’ physical health but also enhance their social–emotional skills and reduce test anxiety. Therefore, schools should consider elevating the frequency of sports as an important component of comprehensive education plans. Secondly, this research indicated that varying levels of social–emotional skills may influence the effectiveness of sports on test anxiety. Educators and mental health professionals should devise more personalized intervention strategies based on different levels of social–emotional skills. Students with lower social–emotional skills can enhance their fundamental social–emotional skills through engagement in sports. Meanwhile, students with higher social–emotional skills might require additional support in other aspects. Finally, it is crucial to integrate the enhancement of social–emotional skills into physical education, as this approach can yield exponential benefits.

Although our study complements existing research and examines the complex relationship among sports participation, test anxiety, and social–emotional skills, there are some limitations that require further examination. First of all, our sample size is all over the world, and there are different cultural backgrounds among them. This also means that the understanding of sports, social–emotional skills, and the ability to withstand test anxiety are different. While controlling for demographic variables, future studies should pay more attention to this point for more detailed research. Secondly, the data are cross-sectional. Although path analysis is sequential, caution is still needed in confirming causality. Studies may have failed to examine changes in the long-term effects of sports on social–emotional skills and test anxiety. Future studies can conduct more in-depth longitudinal studies to confirm the causal relationship. Third, social–emotional skills are a multidimensional and complex concept, encompassing various skills. To prevent collinearity, we adopted a standardized and summative approach to examine social–emotional skills as holistic competencies. Our study conducted an exploratory analysis and initially delineated the role of social–emotional abilities in sports participation and test anxiety. However, for a more profound understanding of the role of each dimension and each skill within it, further in-depth research is necessary.

## 5. Conclusions

Social–emotional skills had a significant mediating effect on the relationship between sports participation and test anxiety. Moreover, social–emotional skills also served as a moderator within this relationship. Specifically, there was a significant attenuation of the negative correlation between sports participation and test anxiety at lower and medium levels of social–emotional skills, with no significant effect observed at higher levels of social–emotional skills. The findings highlight that sports participation and social–emotional skills play a composite role in alleviating test anxiety. By providing a more comprehensive perspective on the relationship between sports participation, social–emotional skills, and test anxiety, this study underscores the importance of incorporating sports participation into strategies aimed at reducing test anxiety. Enhancing social–emotional skills through sports offers a detailed approach to designing effective educational and psychological interventions, ultimately supporting student mental health.

## Figures and Tables

**Table 1:** Descriptive information of the studied sample.

		N (%)	Total ^b^
Cohort	Older (15 years old)	29,798 (48.9%)	60,985
	Younger (10 years old)	31,187 (51.1%)	
Site	Ottawa	5440 (8.9%)	60,985
	Houston	6434 (10.6%)	
	Bogota	6771 (11.1%)	
	Manizales	6757 (11.1%)	
	Helsinki	5482 (9%)	
	Moscow	6792 (11.1%)	
	Istanbul	5869 (9.6%)	
	Daegu	6334 (10.4%)	
	Sintra	3860 (6.3%)	
	Suzhou	72.46 (11.9%)	
Gender	Male	29,863 (48.9%)	60,498
	Female	30,635 (50.2%)	
Sports participation	Yes	35,452 (58.1%)	57,979
	No	22,527 (36.9%)	
Socio-economic status ^a^	M(SD)	0.226 (0.986)	59,583
	Range	−3.75, 3.75	

Note. ^a^: Socio-economic status is a composite index based on data from parental education, parental occupation, and home possessions derived as factor scores from principal component analyses [29]; ^b^: excluding missing data.

**Table 2:** Descriptive statistics and correlations among test anxiety, social–emotional skills, and sports participation (N = 60,874).

	M	SD	1. Test Anxiety	2. Social–Emotional Skills	3. Sports Participation
1	52.947	11.035	1		
2 ^a^	0.000	11.292	−0.166 **	1	
3 ^b^			−0.070 **	0.177 **	1

Note: ^a^: Sum of standardized social-emotional skills. ^b^: The categorical variable forsports participation (N = 57,967) records 22,527 non-participants and 35,452 participants. ** *p* < 0.01.

**Table 3:** Effects of mediation analysis.

	Estimate	SE	LLCI	ULCI	Effect
Total	−0.081	0.009	−0.099	−0.063	1
Direct	−0.029	0.009	−0.047	−0.011	35.80%
Indirect	−0.052	0.002	−0.056	−0.048	64.20%

**Table 4:** Path of moderated analysis (N= 56,996).

Regression Equation		Fitting Index			Significance of Regression Coefficient			
Results	Predictors	*R* ^2^	*F*	Effect	*t*	*p*	LLCI	ULCI
Y	Intercept	0.061	245.668 ***	0.022	1.224		−0.013	0.058
	X			−0.027	−2.934	0.003 **	−0.045	−0.009
	W			−0.016	−25.877	***	−0.017	−0.015
	X × W			0.002	2.649	0.008 **	0.0005	0.004

Note: Y, test anxiety; W, social–emotional skills; X, sports participation. Covariates: gender, cohort, socio-economic status, site. ** *p* < 0.01; *** *p* < 0.001.

**Table 5:** Direct effects and indirect effects at different levels of social–emotional skills.

Social–Emotional Skills		Effect	SE	t	p	LLCI	ULCI
Low	−11.352	−0.050	0.012	−4.134	***	−0.074	−0.026
Medium	0.000	−0.027	0.009	−2.934	0.003 **	−0.045	−0.009
High	11.352	−0.004	0.013	−0.277	0.782	−0.030	0.022

Note: ** *p* < 0.01; *** *p* < 0.001.

## Data Availability

This study relies on the analysis of freely accessible secondary data, available online at https://www.oecd.org/education/ceri/social-emotional-skills-study/ (accessed on 5 February 2024).
